# Decreased Risk in the Pancreatic Cancer With History of Hay Fever: A Meta-Analysis

**DOI:** 10.3389/fpubh.2020.551490

**Published:** 2020-10-06

**Authors:** Guannan Wang, Zhiwei Xu, Jie Zhu, Jiayu Ren, Mina Chen, Guijuan He, Beibei Yu

**Affiliations:** ^1^Blood Purification Center, Ningbo Medical Center, Lihuili Hospital, Ningbo, China; ^2^Department of Critical Care Medicine, Ningbo Medical Center, Lihuili Hospital, Ningbo, China; ^3^Department of Hepato-Biliary-Pancreatic Surgery, Ningbo Medical Center, Lihuili Hospital, Ningbo, China; ^4^Department of Cardiology, Ningbo Medical Center, Lihuili Hospital, Ningbo, China; ^5^Department of Endocrinology and Respiratory Medicine, Ningbo Medical Center, Lihuili Hospital, Ningbo, China; ^6^School of Nursing, Zhejiang Chinese Medical University, Hangzhou, China; ^7^Outpatient Dressing Room, The Affiliated Xiangshan Hospital of Wenzhou Medical University, Xiangshan, China

**Keywords:** pancreatic cancer, allergy, immune surveillance, hay fever, meta-analysis

## Abstract

**Background:** An increasing incidence of pancreatic cancer has been observed worldwide over the last few decades. Previous reports suggested that hay fever, a common allergic disease, may function in pancreatic cancer. Data on hay fever as a risk or protective factor for pancreatic cancer was controversial in several case–control reports. So, we here did a meta-analysis on published studies to evaluate the association of hay fever and the risk of pancreatic cancer.

**Methods:** A comprehensive literature search was performed through public databases. The association between hay fever and pancreatic cancer was evaluated by odds ratios (ORs) and 95% confidence intervals (CIs). The Cochran's Q test and I^2^ index were used to evaluate heterogeneity.

**Results:** We included 8 population-based case–control studies involving 10,454 participants from 1986 to 2014. A history of hay fever was associated with a decreased risk of pancreatic cancer (OR, 0.57; 95% CI, 0.50–0.64, *P* < 0.00001) through fixed effect model.

**Conclusion:** The result of our study suggested that hay fever may significantly decrease the risk of pancreatic cancer.

## Introduction

With stealthy onset and no typical symptoms, pancreatic cancer lacks appropriate diagnosis and sensitive treatment, whose five-year survival rate rests only around 9%, while fatality rate ranks seventh among the cancer-related deaths worldwide (1). Although some certain risk factors have been identified, like tobacco smoking, age, obesity, alcohol abuse, non-O blood group, and chronic pancreatitis (2), the etiology of pancreatic cancer has not yet been fully elucidated. For the primary prevention of pancreatic cancer, more risk factors need to be identified.

Severe autoimmune conditions, such as type I diabetes mellitus, rheumatoid arthritis and systemic lupus erythematosus have been found to be associated with pancreatic cancer (3–5). However, little is well known about the role of subtle immunodeficiency in the development of pancreatic cancer. Hay fever is a hypersensitivity disorder of the immune system occurring in response to pollen. Several studies have suggested that the overall incidence of cancer is lower in individuals with a history of hay fever (6, 7).

Several studies have described the relationship between pancreatic cancer and allergy. However, the relationship between pancreatic cancer and hay fever, which is an important type of allergy, was not consistently reported. We, therefore, did a meta-analysis with all published case–control studies to quantitate the association between hay fever and pancreatic cancer.

## Methods

This study was performed and prepared in accordance with the guidelines proposed by Cochrane Collaboration in the Cochrane Handbook for Systematic Reviews of Interventions (http://www.cochranehandbook.org) and the Preferred Reporting Items for Systematic Reviews and Meta-analyses (PRISMA) statement (8).

### Search Method and Inclusion/Exclusion Criteria

A literature search through the PubMed, Web of Science, Cochrane Library, Science Direct, Wiley Online Library, Chinese National Knowledge Infrastructure, and Wanfang Data Resource databases and Google Scholar through to February 17, 2020. The search was conducted by two authors independently. The terms used in the search were “hay fever” and “pancreatic cancer.” The title and abstract of each article were reviewed. Moreover, full-text articles were selected according to the inclusion criteria and the reference lists of the selected papers were also searched. Studies that met the following criteria were included: (a) report original data on the association between hay fever and pancreatic cancer from epidemiological studies; (b) consist of a human case–control study; (c) been written in English; (d) include patients meeting the diagnostic criteria for pancreatic cancer and hay fever; and (e) offer sufficient data for calculating odds ratios (ORs) and their 95% confidence intervals (CIs). The following were exclusion criteria: (a) duplicate data of previous studies; (b) studies not based on a case–control design; and (c) abstracts, comments, reviews, posters, and editorials. Any disagreements regarding exclusion or inclusion of a study resolved the dispute by a joint review with other authors. Only the most recent studies were selected when the reduplicative study produced several publications.

### Data Extraction

The extracted data included the name of first author, publication year, country of origin, ethnicity, matching conditions, and number of subjects with hay fever. Extracted information was entered into a database.

### Quality Assessment

All studies included were assessed methodological quality from three aspects (selection, comparability, and exposure) according to the Newcastle-Ottawa scale (NOS) criteria (9). Scores range from zero (low) to nine stars (high). Articles scoring ≥5 was considered “high quality.”

### Statistical Analysis

The primary result measurements were OR with 95% confidence interval (CI) of developing pancreatic cancer in patients with a history of hay fever. The OR is a measure of association between exposure (hay fever) and an outcome (pancreatic cancer). The OR represents the odds that an outcome will occur given a particular exposure, compared to the odds of the outcome occurring in the absence of that exposure. Cochran's Q test and *I*^2^ index basing on a chi-square distribution were performed to assess heterogeneity. *I*^2^ ranges from 0 to 100%. Low, moderate, large, and extreme heterogeneity corresponded to 0–25, 25–50, 50–75, and 75–100%, respectively (10). If the result of the Q test was *P* > 0.01 and I^2^ < 50%. The Mantel–Haenszel method is used to estimate the pooled odds ratio, assuming a fixed-effects model. Otherwise, ORs were pooled according to the random-effect model (DerSimonian and Laird). If there was significant heterogeneity among the pooled studies, sensitivity analysis for the overall effect was performed by sequentially omitting one case–control study at a time to assess the stability of the results. Additionally, the publication bias was investigated with Egger's test and Begg's test and funnel plot. In the forest plots, OR values <1 suggested a decreased risk, whereas OR values > 1 suggested an increased risk of cases among individuals with a history of hay fever. The statistical analyses were performed using the STATA 15.1 software (Stata Corporation, Texas, USA), Review Manager 5.0.24 (The Nordic Cochrane Centre, Denmark).

## Result

### Literature Search

**Figure 1:**
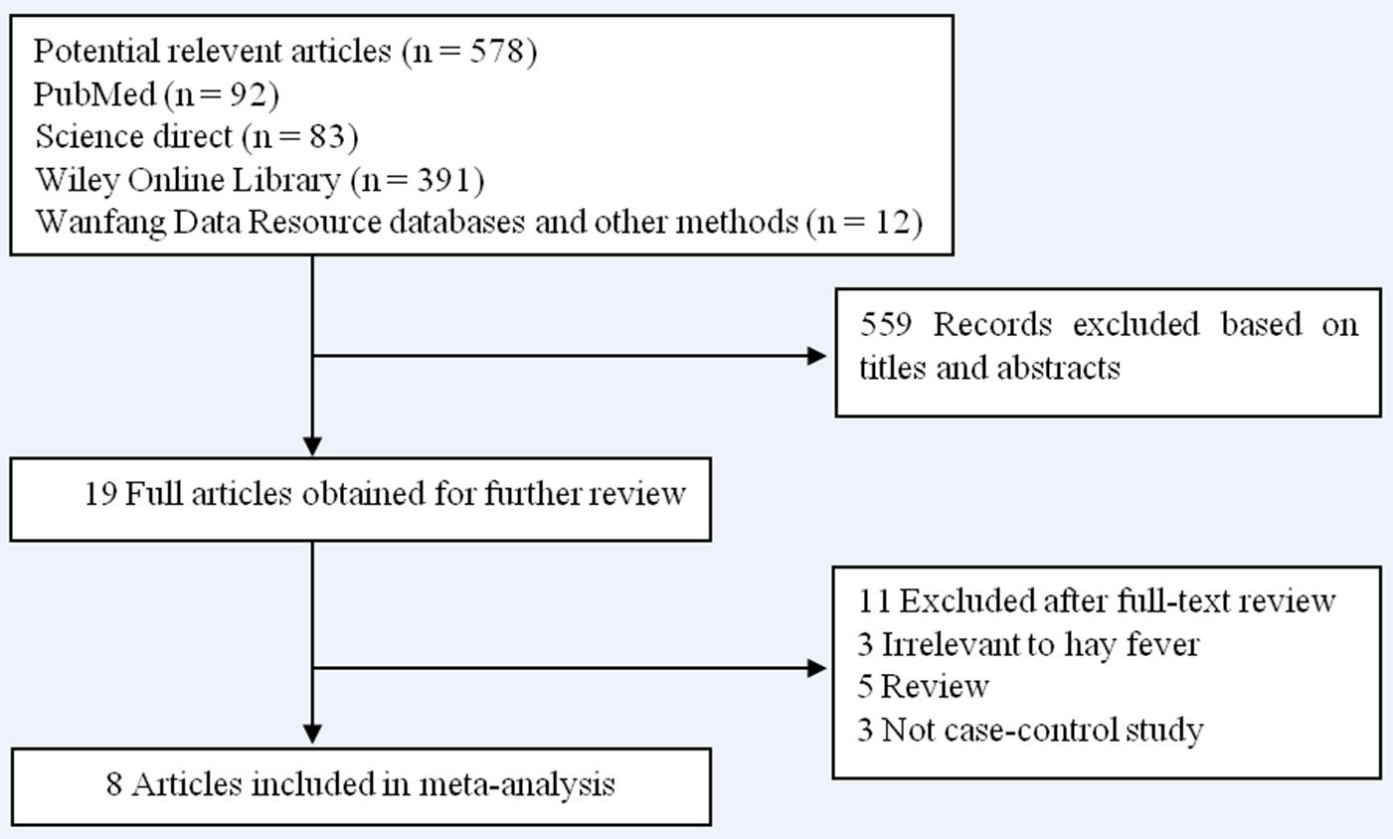
Flow diagram of the literature search and selection.

A total of 578 articles were identified during our initial search. After a careful review, 559 articles which did not pertain to the association between hay fever and the risk of pancreatic cancer were excluded. The remaining 19 articles were retrieved for full-text review. A further 11 articles were excluded (3 were irrelevant to hay fever, 5 were reviews, and 3 were not case–control studies). No additional articles were determined through searching the reference lists of the 19 articles. Finally, 8 case–control studies (11–18) were included in our meta-analysis. The results of the literature search are shown in Figure 1.

### Characteristics of the Studies

The main characteristics of the 8 studies included in our analysis are listed in Table 1 and corresponding quality scores are given in Supplementary Table 1. The time of diagnosis of the cases was between 1976 and 2012. A total of 3,494 pancreatic cancer patients and 6,960 controls involved populations form North America, Europe, and Australia were included in our analysis. To identify the history of hay fever, 3 studies adopted interviewing with questionnaires and reviewing medical records, and the other 5 studies only interviewed with questionnaires. In all studies, the controls were matched to the patients by age and gender and 8 studies were adjusted by region.

**Table 1:** Characteristics of the studies included in the meta-analysis.

**References**	**Location**	**Ethnicity**	**Matching conditions**	**Case**		**Control**	
				n*/n	**Male/female**	n*/n	**Male/female**
Mack et al. (11)	Los Angeles	North America	Age, gender, race, education and birthplace	26/490	282/208	46/490	282/208
Jain et al. (13)	Toronto	North America	Age, gender, race and birthplace	6/249	141/108	35/505	270/235
Silverman et al. (14)	Atlanta, Detroit, New Jersey	North America	Age, gender, race and birthplace	58/484	-	403/2099	-
Olson et al. (16)	New York	North America	Age, gender, education and smoking	90/405	205/200	77/212	88/124
Eppel et al. (15)	Ontario	North America	Age, gender, race and birthplace	56/276	151/125	107/378	176/137
Anderson et al. (12)	Ontario	North America	Age, gender, race and birthplace	86/422	233/189	107/312	176/136
Maisonneuve et al. (17)	Australia, Canada, the Netherlands, Poland	North America, Europe, Australia	Age, gender, race and birthplace	29/823	456/367	97/1679	858/821
Cotterchio et al. (18)	Ontario	North America	Age, gender, race and birthplace	92/345	175/170	464/1285	680/605

n*, the number of people with a history of hay fever; n, total number.

*OR, odds ratio; 95% CI, 95% confidence interval*.

### Outcome Results

**Figure 2:**
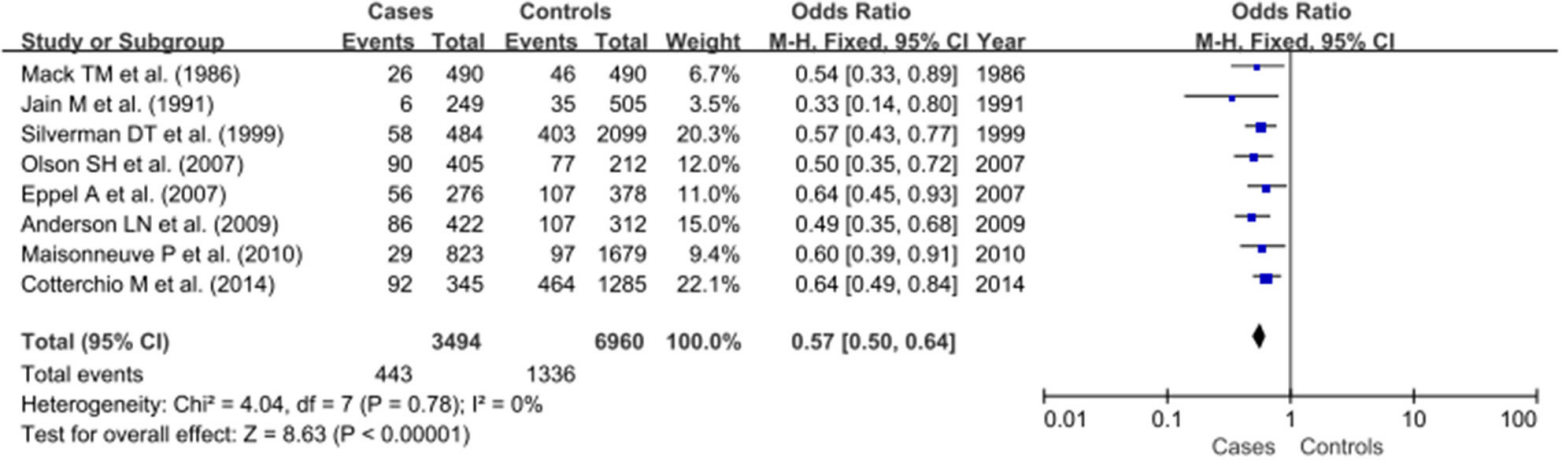
Meta- analysis for the association of hay fever with pancreatic cancer.

As no heterogeneity was detected (*P* > 0.05 and I^2^ < 50%), the fixed-effect model was applied for further meta-analysis. The pooled OR indicated a significant protective effect for hay fever against pancreatic cancer (OR: 0.57, 95% CI, 0.50–0.64, *P* < 0.00001; Figure 2).

### Sensitivity Analysis and Publication Bias

**Figure 3:**
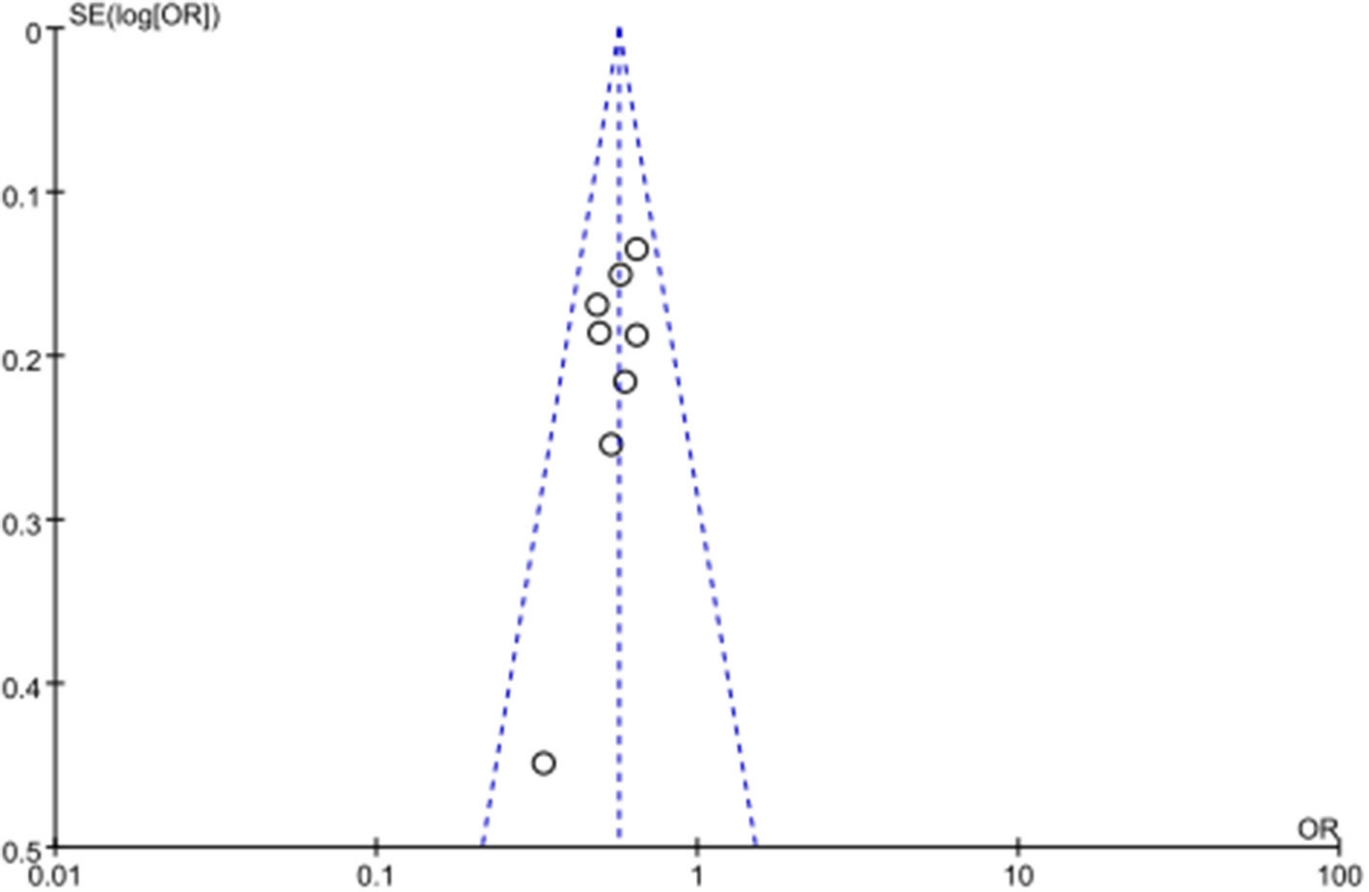
Begg's funnel plot of publication bias. SE, standard error; OR, odds ratio.

As no severe heterogeneity was observed and eligible studies were limited, sensitivity analysis was not performed. No significant publication bias was observed in all above genetic models via Begg's funnel plot and Egger's test (all *P* > 0.05, data not shown). The symmetrical distribution of studies in the funnel plot indicated the absence of any potential publication bias (Figure 3).

## Discussion

Results from our meta-analysis support evidence of a lower risk of pancreatic cancer among people with a history of hay fever (OR: 0.57, 95% CI, 0.50–0.64, *P* < 0.00001). Similar associations were also reported between hay fever and risk of cancer of the colon and rectum, lung, breast, and melanoma (19–22). Additionally, based on the 2015 National Health Interview Survey database, a retrospective study found that a personal history of hay fever was significantly inverse associated with overall cancer diagnoses in univariate and multivariate regression (7). Meanwhile, some studies were not able to identify such risk reduction (19), suggesting that the association between hay fever and cancer is complex.

The role of hay fever on the risk of developing pancreatic cancer has been studied for decades, but the mechanism remains unclear. Up to date, several hypotheses have been presented and enhanced immune surveillance was the most widespread theory to explain the inverse association between hay fever with cancer (23). The hyperactive immune system may play an important role in tumor prevention in eliminating neoplastic and preneoplastic cells through an IFN-γ-dependent mechanism. This hypothesis is enhanced by the presence of higher IgE levels among patients (24) and allergy-associated genetic variants in IL4 and IL4Ra are inversely associated to pancreatic cancer (16). Thus, the occurrence of hay fever might be a surrogate marker, which has an increased ability to recognize and destroy malignant cells in the immune system. In our meta-analysis, hay fever was associated with a reduced risk of pancreatic cancer, which further supports the immune surveillance hypothesis.

Due to certain limitations, the result of our meta-analysis should be interpreted with caution. First, in most of the studies, the history of hay fever was obtained from interview by questionnaire or telephone, which may be inaccurate or subject to bias (18). Second, the majority of data were obtained from the North American population, with Asian individuals being insufficiently represented. Third, confounding factors including region of residence, age, and gender were controlled in most of the included studies, but other factors, such as individual lifestyle, genetic characteristics, and other immune-related diseases cannot be completely ruled out. Meanwhile, there were also several strengths in our meta-analysis. Firstly, the number of included controls and cases was sufficiently large to obtain a reliable relationship between hay fever and pancreatic cancer. Secondly, all studies included in our meta-analysis have no significant heterogeneity or publication bias was detected, so that we can minimize the potential information bias between controls and cases.

In conclusion, our study demonstrated that hay fever is inversely associated with the incidence of pancreatic cancer. Because of the limited epidemiological studies, however, future additional large prospective studies are required to elucidate the association between hay fever and pancreatic cancer.

## Data Availability Statement

The raw data supporting the conclusions of this article will be made available by the authors, without undue reservation.

## Author Contributions

GH and BY conceived and designed the experiments and revised the manuscript. JZ, JR, and MC were involved in data analysis. GW and ZX drafted the manuscript and contributed equally to this work. All authors were involved in the revision of the manuscript.

## Conflict of Interest

The authors declare that the research was conducted in the absence of any commercial or financial relationships that could be construed as a potential conflict of interest.
