# Supplementary material for: Decreased Risk in the Pancreatic Cancer With History of Hay Fever: A Meta-Analysis
Source: Front Public Health. 2020 Oct 6;8:551490. doi: 10.3389/fpubh.2020.551490 (PMC7574341; doi:10.3389/fpubh.2020.551490)
Supplement: Supplementary file 1 [file Table_1.doc]

| Table S1. Newcastle-Ottawa scale (NOS) scores of the studies included in the meta-analysis. | | | | | | | | | |
| --- | --- | --- | --- | --- | --- | --- | --- | --- | --- |
| Study | Selection |  |  |  | Comparability | Exposure |  |  | NOS score |
|  | Adequate definition of cases | Representati-veness of the cases | Selection of controls | Definition of controls | Control for important factor | Ascertainment of exposure | Same method of ascertainment for cases and controls | Non-response rate |
| Mack TM et al. (1986) | ☆ | ☆ | ☆ | ☆ | ☆☆ | ☆ | ☆ | - | 8 |
| Jain M et al. (1991) | ☆ | ☆ | ☆ | ☆ | ☆☆ | ☆ | ☆ | ☆ | 9 |
| Silverman DT et al. (1999) | ☆ | ☆ | ☆ | ☆ | ☆ | ☆ | ☆ | ☆ | 8 |
| Olson SH et al. (2007) | ☆ | ☆ | - | ☆ | ☆☆ | ☆ | ☆ | - | 7 |
| Eppel A et al. (2007) | ☆ | ☆ | ☆ | ☆ | ☆☆ | ☆ | ☆ | ☆ | 9 |
| Anderson LN et al. (2009) | ☆ | ☆ | ☆ | ☆ | ☆☆ | ☆ | ☆ | - | 8 |
| Maisonneuve P et al. (2010) | ☆ | ☆ | - | ☆ | ☆☆ | ☆ | ☆ | - | 7 |
| Cotterchio M et al. (2014) | ☆ | ☆ | ☆ | ☆ | ☆☆ | ☆ | ☆ | ☆ | 9 |
|  |  |  |  |  |  |  |  |  |  |
